# Improvement in B_1_^+^ Homogeneity and Average Flip Angle Using Dual-Source Parallel RF Excitation for Cardiac MRI in Swine Hearts

**DOI:** 10.1371/journal.pone.0139859

**Published:** 2015-10-05

**Authors:** Michael Schär, Haiyan Ding, Daniel A. Herzka

**Affiliations:** 1 Russell H. Morgan Department of Radiology and Radiological Science, Division of Magnetic Resonance Research, Johns Hopkins University, Baltimore, Maryland, United States of America; 2 Clinical Science MRI, Philips Healthcare, Cleveland, Ohio, United States of America; 3 Biomedical Engineering, Johns Hopkins School of Medicine, Baltimore, Maryland, United States of America; 4 Biomedical Engineering, Tsinghua University, Beijing, China; University of Chicago, UNITED STATES

## Abstract

Cardiac MRI may benefit from increased polarization at high magnetic field strength of 3 Tesla but is challenged by increased field inhomogeneity. Initial human studies have shown that the radiofrequency (RF) excitation field (B_1_^+^) used for signal excitation in the heart is both inhomogeneous and significantly lower than desired, potentially leading to image artifacts and biased quantitative measures. Recently, multi-channel transmit systems have been introduced allowing localized patient specific RF shimming based on acquired calibration B_1_^+^ maps. Some prior human studies have shown lower than desired mean flip angles in the hearts of large patients even after RF shimming. Here, 100 cardiac B_1_^+^ map pairs before and after RF shimming were acquired in 55 swine. The mean flip angle and the coefficient of variation (CV) of the flip angle in the heart were determined before and after RF shimming. Mean flip angle, CV, and RF shim values (power ratio and phase difference between the two transmit channels) were tested for correlation with cross sectional body area and the Right-Left/Anterior-Posterior ratio. RF shimming significantly increased the mean flip angle in swine heart from 74.4±6.7% (mean ± standard deviation) to 94.7±4.8% of the desired flip angle and significantly reduced CV from 0.11±0.03 to 0.07±0.02 (p<<1e-10 for both). These results compare well with several previous human studies, except that the mean flip angle in the human heart only improved to 89% with RF shimming, possibly because the RF shimming routine does not consider safety constraints in very large patients. Additionally, mean flip angle decreased and CV increased with larger cross sectional body area, however, the RF shimming parameters did not correlate with cross sectional body area. RF shim power ratio correlated weakly with Right-Left/Anterior-Posterior ratio but phase difference did not, further substantiating the need for subject specific cardiac RF shimming.

## Introduction

MRI scanners at a static magnetic field strength of 3 Tesla (T) became available for cardiac MRI in 2001. Compared to 1.5 T, the available bulk magnetization at 3 T increases linearly with the field strength offering higher signal-to-noise ratio (SNR) that can be traded for higher spatial resolution and / or faster imaging, both highly desired prospects in cardiovascular magnetic resonance (CMR). However, clinical CMR is still mainly performed at 1.5 T. Challenges with CMR at 3 T are usually listed as 1) increased susceptibility-induced main magnetic field (B_0_) inhomogeneities, 2) increased radiofrequency (RF) power deposition for a given flip angle, and 3) increased inhomogeneities of the RF excitation field (B_1_^+^) used for signal excitation [1–3]. In this study we will focus on the B_1_^+^ field.

Conventional clinical MRI scanners up to a magnetic field strength of 3 T use an integrated birdcage quadrature coil to generate the B_1_^+^ field. Both numerical simulations [4] and measurements [5] have shown that the B_1_^+^ field produced by a quadrature coil in the heart is more inhomogeneous at 3 T compared to 1.5 T. Using the saturated double-angle method (SDAM) [6] to acquire cardiac B_1_^+^ maps, Sung et al. observed a flip angle variation ranging from 31% to 66% of the applied flip angle over the entire left ventricular volume in humans [7]. In terms of actually applied flip angles, they reported a range of values from 34° to 63° across the left ventricle for a nominal flip angle of 60°. This not only demonstrates that the B_1_^+^ field over the left ventricle is inhomogeneous but additionally, and maybe more importantly, that the flip angle (RF power setting) is on average about 20% lower than the one requested. This finding has been reproduced on another vendor’s 3 T scanner where the average B_1_^+^ over both ventricles was 27% lower than desired [8]. Since these are average values, the flip angle in the heart will be close to what is desired in some patients but even lower in others. Low flip angles will lead to local signal reduction, failure of magnetization preparation pulses, and eventually to artifacts and biased quantitative measures. Setting the RF power based on the acquired B_1_^+^ maps enabled correct average flip angles within 1% of the desired angle while the variation over the heart remained the same [8]. Recently, it has been shown that multi-channel transmit systems can be used to reduce the B_1_^+^ variation over the heart by the use of RF shimming [9–13]. In a total of 64 human subjects [9–11], the coefficient of variation (CV, standard deviation divided by mean) of B_1_^+^ over the heart in a single imaging slice improved by 43% from 0.12 to 0.07 (Table 1).

**Table 1:** Average flip angle and CV of the flip angle in the heart before and after RF shimming as reported in the literature in humans and results from this study in swine.

	n, species	B_1_^+^ map orientation	pre RF-shim flip angle (% of desired)	post RF-shim flip angle (% of desired)	CV pre RF-shim	CV post RF-shim
Mueller et al. [9]	13, humans	transverse	72.8±4.0	91.9±3.3	0.12±0.08	0.07±0.03
Krishnamurti et al. [10]	37, humans	transverse	81.2±13.3	88.5±15.2	0.13±0.03	0.07±0.02
Jia et al. [11]	14, humans	short axis view	76.7±9.7	85.4±9.5	0.10±0.05	0.07±0.02
Jogiya et al. [12]	30, humans	transverse	74.2±8.3	91.1±1.9	n.a.	n.a.
Weighted average excluding this work	94 for FA; 64 for CV		77.1	89.3	0.12	0.07
**This work**	**100, swine**	**transverse**	**74.4±6.7**	**94.7±4.8**	**0.11±0.03**	**0.07±0.02**

However, in 94 human subjects [9–12] the error in the average flip angle over the heart was only reduced by 53% from 23% under-tipping to 11% under-tipping (Table 1). While the underlying physics constrains how much the B_1_^+^ variation can be improved, there is no reason for the average flip angle over the heart not to be at the desired value as has been shown in [8].

In this work, RF shimming of the heart using a two-channel multi-transmit system is evaluated in swine. B_1_^+^ maps in the heart are acquired prior to and post RF shimming to quantify improvement in B_1_^+^ variation and average flip angle over the heart. A special focus is placed on the average flip angle / RF power setting.

## Materials and Methods

### Data acquisition

All animal studies were approved by the Institutional Animal Care and Use Committee (protocol SW14M263). The performance of cardiac RF shimming was studied in swine being imaged as part of other studies. Those studies included high-resolution quantitative imaging such as T2-mapping [14] after acute and chronic myocardial infarction or radio-frequency ablation and high-resolution delayed enhancement imaging. At the end of those studies, the animals were euthanized by barbiturate overdose (Pentobarbital 100mg/kg IV) followed by IV potassium chloride. During imaging, animals were anesthetized with isoflurane 1–2.5%, along with 100% oxygen, and mechanically ventilated. Studies were performed on a 3 T MR scanner (Achieva R3.2, Philips Healthcare, Best, Netherlands) equipped with an integrated two-channel multi-transmit body coil. Image processing and statistical analysis were implemented in MATLAB (The MathWorks, Natick, Massachusetts).

**Fig 1:**
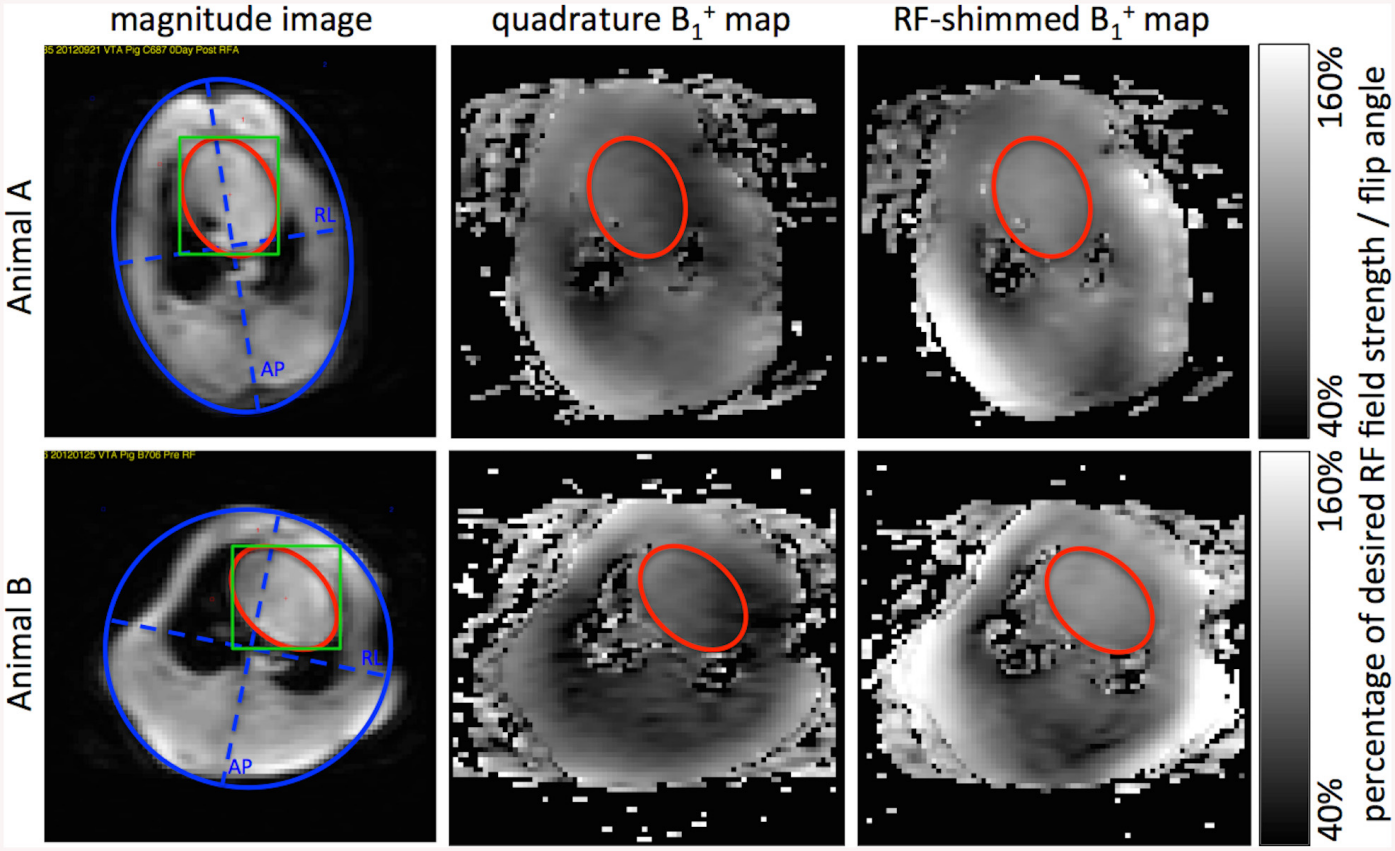
Representative B_1_^+^ maps and ROI placement. Axial magnitude images (1^st^ column) through the center of the heart are used to select the heart (red) and cross sectional body area (blue) elliptical ROIs. The scanner routine was used to calculate RF-shim values localized to a manually drawn rectangle (green). Corresponding B_1_^+^ maps acquired in quadrature mode pre-RF shimming and RF-shimmed are shown in the 2^nd^ and 3^rd^ columns, respectively. Data from two different animals A and B are shown in the two rows. Lengths of RL and AP were defined as the dash blue lines for RL/AP ratio calculation.

Single-slice B_1_^+^ maps were acquired pre and post RF shimming from the hearts of 64 consecutive swine. Data from 9 were not considered due to either missing pre RF shimming map or log files. Some animals were imaged on multiple occasions a few hours to 180 days apart leading to n = 100 B_1_^+^ map pairs. Two animals were imaged 5 times, one 4 times, six 3 times, 22 twice, and 24 once. B_1_^+^ maps were acquired with the SDAM [6] method using the following imaging parameters: ECG triggering, repetition time one heart beat, echo time = 3.3 ms, saturation delay 500 ms, flip angles 60° and 120°, spoiled gradient echo with EPI factor 11, spectrally selective adiabatic inversion recovery fat suppression with an inversion delay of 180 ms, field of view 320 x 320 mm^2^, slice thickness 10 mm, acquired voxel size 5 x 11 mm^2^, reconstructed voxel size 4 x 4 mm^2^, integrated body coil for signal reception, breath-hold duration 7 s. The imaging slice was acquired in transverse orientation through the center of the heart. The two transmit channels were set to quadrature mode (same amplitude and 90° phase shift) for the pre B_1_^+^ map. A calibration B_1_^+^ map was acquired after the pre B_1_^+^ map. For the calibration B_1_^+^ map two maps were acquired in a 12 s breath-hold, one for each transmit channel. Because excitation was performed with only one transmit channel at a time for the calibration B_1_^+^ map, the duration of the excitation pulse was doubled. For both pre and calibration B_1_^+^ map acquisitions, the RF power was determined using the vendor’s standard non-localized calibration. For RF shimming, the system-provided routine was used to calculate both the power ratio and phase difference between the two transmit channels, and the RF power required to achieve the desired flip angle. The RF shim routine minimized the flip angle distribution based on the calibration B_1_^+^ map within a user-selected rectangle. In this study this rectangle was placed over the heart (green box in Fig 1). The calculated RF power, power ratio, and phase difference were then applied for the acquisition of the post B_1_^+^ map. The applied RF shim values, power ratio and phase difference, were recorded and any messages concerning RF power limitations were explicitly noted.

### Data analysis

Two elliptical regions of interest (ROI) were drawn on magnitude images of the B_1_^+^ maps to select the heart and the cross sectional body area (Fig 1). The two main axes of the body ROI were used to determine the Right-Left (RL)/Anterior-Posterior (AP) ratio. The heart ROI was copied to the pre and post B_1_^+^ maps to determine 1) the mean achieved flip angle within the ROI, as a percentage of the intended flip angle, and 2) the CV of the flip angle defined as the ratio of the standard deviation to the mean within the ROI.

The pre and post RF shimming mean achieved flip angle, pre and post CV of the flip angle, and the two RF shim values, power ratio and phase difference, were tested for linear correlation with both RL/AP ratio (as suggested in [10]) and cross sectional body area using the MATLAB function fitlm. An α value of 0.05 was used for each family of statistical tests (two families each with 6 tests). Hence, a modified Bonferroni correction was applied to individual test p-values to determine statistical significance (S) or lack thereof (NS) [15]. Mean flip angle and CV were also compared before and after RF shimming using a paired Student’s t-Test using Microsoft Excel. A p-value smaller than 0.05 was considered significant for those tests.

## Results

**Fig 2:**
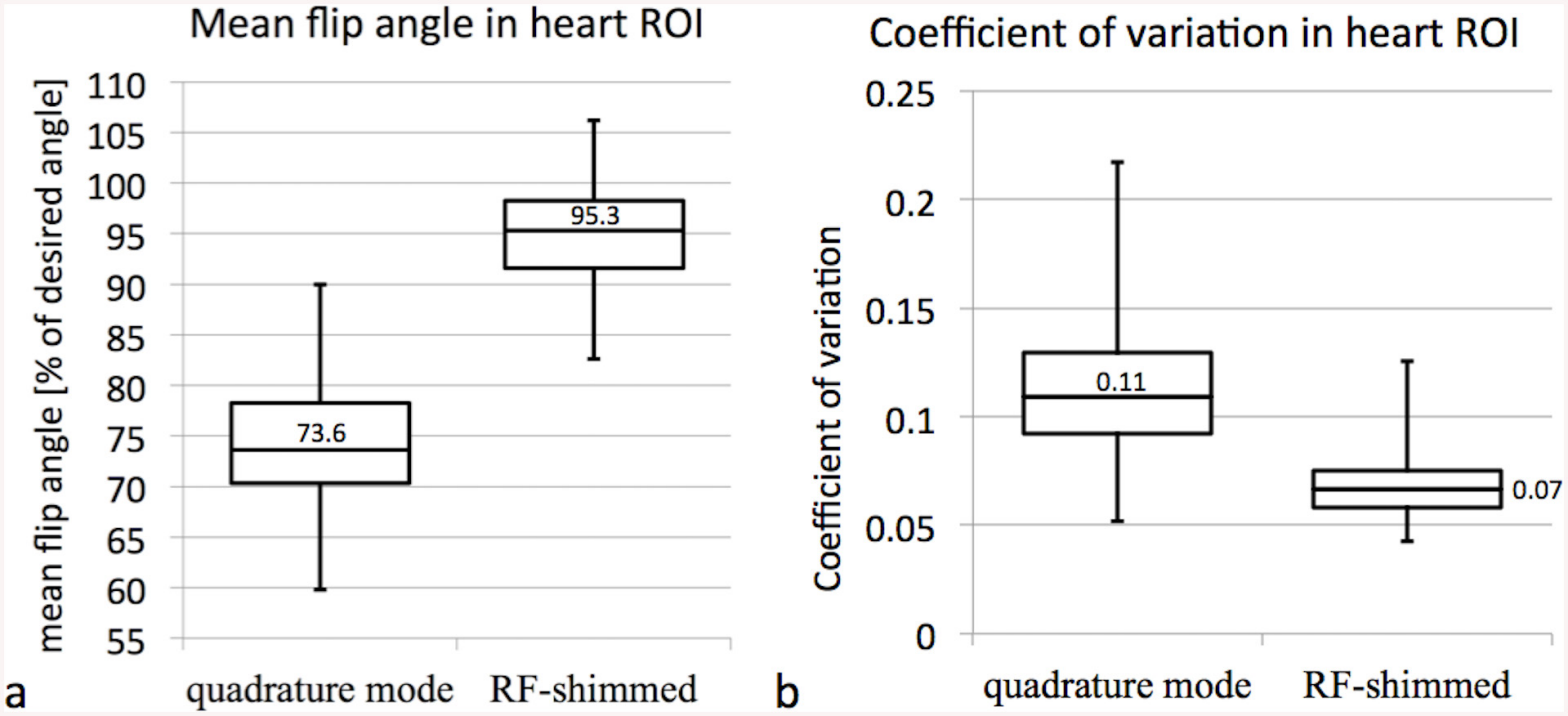
Mean and CV of the flip angle with and without RF shimming. Box plots (a) of the mean flip angle and (b) the CV of the flip angle in the heart with either conventional quadrature mode or with subject specific RF shimming.

RF shimming and the acquisition of pre and post B_1_^+^ maps were successful in all n = 100 attempts. Measured mean and CV flip angle pre and post RF shimming, RF shim values, cross sectional body area, and AP/RL ratio are provided as supplementary information (S1 Dataset). The mean flip angle in the heart ROI with RF transmission using the standard quadrature mode was 74.4±6.7% (mean ± standard deviation) of the desired flip angle. RF shimming significantly improved the mean flip angle in the heart to 94.7±4.8% of the desired flip angle (p<<1e-10, Fig 2a). This corresponds to a 79% reduction of the error in the average flip angle in swine compared to the 53% reduction reported previously in humans [9–12]. The CV of the flip angle in the heart ROI significantly improved with RF shimming from 0.11±0.03 to 0.07±0.02 (p<<1e-10, Fig 2b).

**Fig 3:**
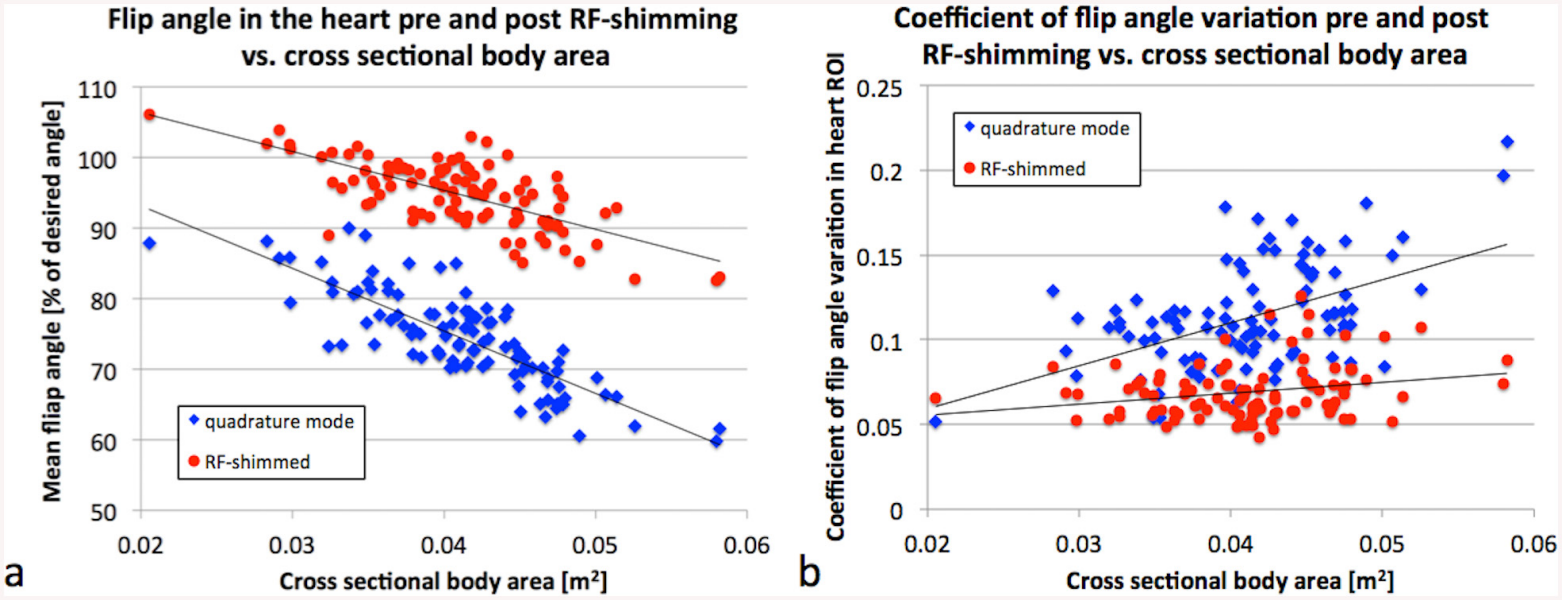
Mean and CV of the flip angle vs. cross sectional body area. Linear correlation (a) of the mean flip angle and (b) the CV of the flip angle in the heart with cross sectional body area. The mean flip angles with RF shimming (red dots) are improved compared to conventional quadrature mode (blue diamonds) in all cases.

In Fig 3a the mean flip angle in the heart is plotted against the cross sectional body area. RF shimming increased the mean flip angle in all cases. The mean flip angle in the heart linearly correlated strongly with the cross sectional body area both pre and post RF shimming with R^2^ = 0.66 (p<<1e-10, S) and R^2^ = 0.49 (p<<1e-10, S), respectively. Fig 3b shows CV in the heart plotted vs. body area. CV correlated with the cross sectional body area moderately pre and weakly post RF shimming with R^2^ = 0.25 (p = 1e-6, S) and R^2^ = 0.06 (p = 0.015, S), respectively. Neither mean flip angle nor CV correlated with AP/RL ratio (p>0.5, NS for all).

**Fig 4:**
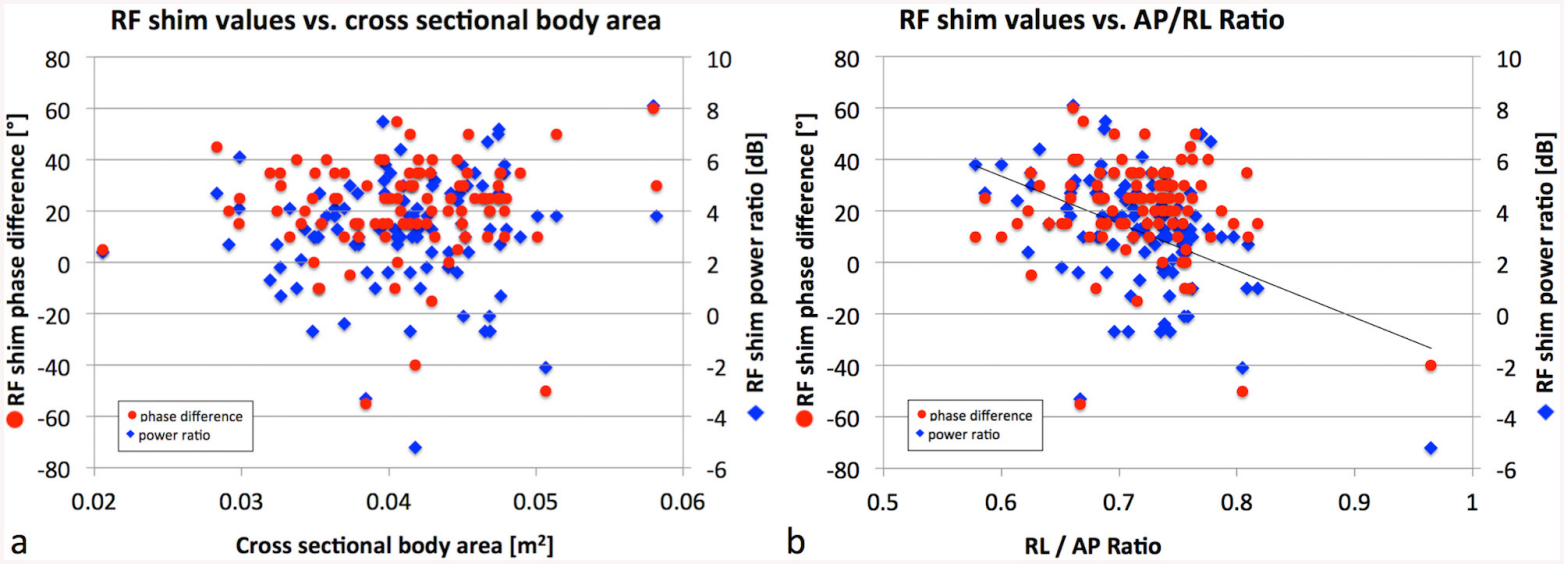
RF shim values vs. cross sectional body area and RL/AP ratio. RF shim values power ratio and phase difference vs. (a) cross sectional body area and (b) RL/AP ratio. They do not correlate with cross sectional body area (R^2^ = 0.01, p = 0.2 and R^2^ = 0.01, p = 0.3, respectively), and phase difference does not correlate with AP/RL ratio (R^2^ = 0.04, p = 0.06), but power ratio weakly correlates with AP/RL ratio (R^2^ = 0.05, p<1e-5).

The RF shim values power ratio and phase difference did not correlate with cross sectional body area (Fig 4a, R^2^ = 0.01, p = 0.2, NS, and R^2^ = 0.01, p = 0.3, NS, respectively). The phase difference did not correlate with AP/RL ratio (R^2^ = 0.04, p = 0.06, NS); however, there was a weak correlation between power ratio and AP/RL ratio (Fig 4b, R^2^ = 0.05, p<1e-5, S). This further demonstrates that optimal RF-shim settings need to be determined based on a calibration scan per subject and cannot be deduced on more simple measures such as cross sectional body size or AP/RL ratio [10].

The RF power was limited in two of the 100 cases. In these two cases the power was reduced to stay within safety constraints leading to a reduced applied flip angle. In one case (cross sectional body area = 0.042 m^2^) the RF power was reduced to keep the measured B_1_^+^ field in the integrated pickup coil of the transmit coil below the safety threshold. In the second case (cross sectional body area = 0.058 m^2^) the RF power was limited by the maximum peak power.

## Discussion

The achievable accuracy and quantitative nature of CMR have led to its broad application in research involving swine. For example, CMR in swine has been critical in basic research of atherosclerosis [16], myocardial infarction [14, 17], for safety testing of devices [18], interventions [19, 20], and contrast agents [21]. Studies such as this one that analyze nonlinearities associated with higher field imaging that can lead to errors in quantitation are of interest. This study represents the first investigation of the RF flip angle distribution in the hearts of swine at 3 T and utilizes a large number of animals to improve measurement reliability.

The results obtained in this study demonstrate that, in a manner similar to human studies, the average flip angle in the heart of swine at 3 T when using the standard quadrature mode was only 74% of the desired flip angle compared to 77% in humans (Table 1). Likewise, the CV of the flip angle in swine heart was 0.11 compared to 0.12 in humans when using quadrature mode. Applying subject specific, localized RF shimming with a two-channel multi-transmit whole body coil significantly reduced CV to 0.07, the same as in previous human studies. Determining the RF power based on the calibration B_1_^+^ maps in combination with RF shimming significantly increased the average flip angle in swine heart from 74% to 95% of the desired flip angle, whereas previous human studies only achieved 89%. Examining Fig 3 in [10] shows that RF shimming in a human patient population increased the average flip angle in the heart in most patients with low body mass index and low body surface area, whereas it decreased for most of the 10 patients with highest body mass index and body surface area. In this study, the average flip angle in the heart increased after RF shimming in all 100 cases, and the RF power was limited due to safety considerations in two of them. One of the two cases was one of the largest animals in the study. Based on this we hypothesize that 1) the average flip angle with RF shimming in previous human studies was <90% of the desired flip angle because the RF power was limited due to safety constraints in large patients, and 2) this could potentially be corrected by modifying the RF shim calculator to consider additional safety constraints instead of only minimizing B_1_^+^ inhomogeneity.

In adult humans, Krishnamurthy et al. found no dependency of either the average flip angle or the CV on the body type of the patients by correlating average flip angle and CV pre and post RF shimming with body mass index, body surface area, and AP/RL ratio [10]. This work confirmed the lack of correlation with AP/RL ratio. However, it is shown that in swine, both average flip angle and CV significantly correlate with cross sectional body area, a measure that has not been tested in humans yet (Fig 3). The correlation is reduced after RF shimming, but still significant. This is of interest because measures such as body type or size could be determined easier and faster compared to the acquisition of a calibration B_1_^+^ map. Despite the correlation of average flip angle and CV, no correlation of the RF shim values (amplitude ratio or phase difference) with cross sectional body area was found (Fig 4a). However, there was a weak correlation between RF shim amplitude ratio and AP/RL ratio (Fig 4b), which was not observed in humans [10]. Despite this weak correlation for the amplitude ratio, the large range in the determined phase difference still indicates the need for subject specific determination of RF shim values, i.e. the acquisition of a calibration B_1_^+^ map in each subject.

In this work, and all the referenced human studies in Table 1, a two-channel multi-transmit system has been used. Using more transmit channels, B_1_^+^ inhomogeneity may be further reduced or B_1_^+^ homogeneity could be traded off to reduce the required RF power as has been shown in other body parts [22].

This is not a human study though the results obtained here do extend to human subjects, as the proportions of the large animals in this work are similar to those of humans. Nevertheless, further studies in humans will be required to demonstrate that patient-specific RF shimming should be used as a standard at 3 T, and even higher field strengths.

## Conclusions

In swine, the average applied flip angle and the CV of the flip angle in the heart are significantly improved with RF shimming compared to standard quadrature mode. Both parameters correlate with cross sectional body area both before and after RF shimming, indicating that the RF shim routine could potentially be further improved for large subjects.

## Supporting Information

S1 Dataset ROI data from all B_1_+ maps. (XLSX)
